# Natural Small Molecules in Breast Cancer Treatment: Understandings from a Therapeutic Viewpoint

**DOI:** 10.3390/molecules27072165

**Published:** 2022-03-27

**Authors:** Md. Rezaul Islam, Fahadul Islam, Mohamed H. Nafady, Muniya Akter, Saikat Mitra, Rajib Das, Humaira Urmee, Sheikh Shohag, Aklima Akter, Kumarappan Chidambaram, Fahad A. Alhumaydhi, Talha Bin Emran, Simona Cavalu

**Affiliations:** 1 Department of Pharmacy, Faculty of Allied Health Sciences, Daffodil International University, Dhaka 1207, Bangladesh; md.rezaulislam100ds@gmail.com (M.R.I.); fahadulislamdiu@gmail.com (F.I.); muniyaakter3012@gmail.com (M.A.); aklima.ph@diu.edu.bd (A.A.); 2 Faculty of Applied Health Science Technology, Misr University for Science and Technology, Giza 12568, Egypt; mohamed.nafady@must.edu.eg; 3 Department of Pharmacy, Faculty of Pharmacy, University of Dhaka, Dhaka 1000, Bangladesh; saikatmitradu@gmail.com (S.M.); rajibjony97@gmail.com (R.D.); 4 Department of Pharmaceutical Science, North South University, Dhaka 1229, Bangladesh; humaira.urmee101@gmail.com; 5 Department of Biochemistry and Molecular Biology, Faculty of Life Science, Bangabandhu Sheikh Mujibur Rahman Science and Technology University, Gopalganj 8100, Bangladesh; sheikhshohag.bmb@gmail.com; 6 Department of Pharmacology and Toxicology, College of Pharmacy, King Khalid University, Abha 62529, Saudi Arabia; kumarappan@kku.edu.sa; 7 Department of Medical Laboratories, College of Applied Medical Sciences, Qassim University, Buraydah 52571, Saudi Arabia; f.alhumaydhi@qu.edu.sa; 8 Department of Pharmacy, BGC Trust University Bangladesh, Chittagong 4381, Bangladesh; 9 Faculty of Medicine and Pharmacy, University of Oradea, 410087 Oradea, Romania

**Keywords:** natural compounds, antitumor, immune-suppressive, breast cancer, combination therapy

## Abstract

Breast cancer (BrCa) is the most common malignancy in women and the second most significant cause of death from cancer. BrCa is one of the most challenging malignancies to treat, and it accounts for a large percentage of cancer-related deaths. The number of cases requiring more effective BrCa therapy has increased dramatically. Scientists are looking for more productive agents, such as organic combinations, for BrCa prevention and treatment because most chemotherapeutic agents are linked to cancer metastasis, the resistance of the drugs, and side effects. Natural compounds produced by living organisms promote apoptosis and inhibit metastasis, slowing the spread of cancer. As a result, these compounds may delay the spread of BrCa, enhancing survival rates and reducing the number of deaths caused by BrCa. Several natural compounds inhibit BrCa production while lowering cancer cell proliferation and triggering cell death. Natural compounds, in addition to therapeutic approaches, are efficient and potential agents for treating BrCa. This review highlights the natural compounds demonstrated in various studies to have anticancer properties in BrCa cells. Future research into biological anti-BrCa agents may pave the way for a new era in BrCa treatment, with natural anti-BrCa drugs playing a key role in improving BrCa patient survival rates.

## 1. Introduction

Breast cancer (BrCa) is considered a global public health concern [1]; it is the second most widely diagnosed cancer and a prominent cause of mortality in women [2]. BrCa has become a lethal disease, and the risk factors connected with it appear to be growing by the day [3]. Many external and endogenous factors can promote BrCa pathology and exacerbate the condition [4]. BrCa affects about one in every eight women at some point in their lives [5]. BrCa is considered the world’s second-highest cause of cancer-related deaths in women. As a result, to reduce the number of BrCa-related mortality, effective BrCa therapies are necessary [6,7].

Furthermore, people of certain races or ethnicities are more likely to develop BrCa. African American women under the age of 40 are twice as likely to develop BrCA as white women of the same age. Females of American, African, and Hispanic heritage can be identified with aggressive and severe types of BrCa [7]. BrCa differences highlight the need for more effective BrCa treatment. Because of its significant prevalence, effective treatments are required to treat BrCa [8]. Surgical procedures, chemotherapy treatments, radiotherapy, and hormone therapy procedures are the most common treatments for BrCa. Even though chemotherapeutic medications are frequently used to treat BrCa, the occurrence of drug resistance, adverse effects, and disease recurrence show that these drugs are inefficient [9,10]. ABCB1, ABCG2, and ABCC1 are three drug efflux genes that have been extensively explored [11]. Chemotherapeutic agents have many side effects, such as neuropathy, constipation, diarrhea, nausea, vomiting, and breathing difficulties. Organic chemical molecules created by living organisms must be substituted for present chemotherapeutic drugs because clinicians require treatments that cure BrCa without significant drug efflux or side effects [12]. Natural medications effectively treat BrCa and have few to no identified side effects. There have also been many studies of natural chemical-based therapies for breast cancer subtypes, for instance, HER-2, Luminal, and Basal-like [13]. In many circumstances, organic drugs are speedier, cheaper, safer, and cause lesser harm when used in the BrCa therapy [10]. Some natural combinations can induce chemo-sensitization and apoptosis [11,12].

This review aims to discuss and evaluate these natural compounds and validate their efficiency compared to the conventional therapeutic methods for treating BrCa.

## 2. Various Subtypes of Breast Cancer

**Figure 1:**
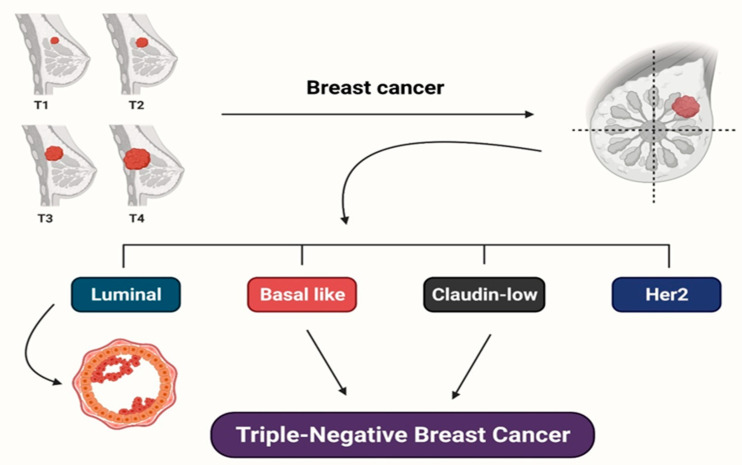
Molecular subtypes of breast cancer and triple-negative breast cancer types are depicted. Overall survival (for breast cancer subtypes) and relapse-free survival (for TNBC subtypes) are used to distinguish the subtypes.

Ductal hyperproliferation is the first stage of tumorigenesis. Several carcinogenic factors can influence the progression of benign or metastatic tumors [14]. Based on genomic profiling of breast tumors, Perou et al. [15,16] classified numerous molecular types of BrCa into the four basic categories of breast tumors (Figure 1). Gene overexpression is generally linked with basal-like and luminal subtypes and associated with long and short survival rates. However, the (human epidermal growth factor receptor 2 (HER2) subordinate type is correlated with epidermal growth factor receptor (EGFR) overexpression, and a collection of specific genes is also associated with short survival. It is worth noting that the exact reasons behind the poor prognosis of basal-like cancers are still unknown. Later, Prat et al. [17] recognized a new-found subtype of claudin-deficient tumors with the considerable representation of cancer stem cell-like characteristics, markers of the epithelial-to-mesenchymal transition, and immune response genes. This subtype has poor to non-existent luminal distinction markers, associated with a lower survival rate [18]. 

Surprisingly, both of these subtypes share a lot of genetic variability and many of the same features as triple-negative breast tumors (TNBCs). TNBCs are distinguished from other tumors by the absence of three biomarkers, namely the estrogen receptor (ER), progesterone receptor (PR), and HER2 proteins. Although Lehmann et al. [19] unveiled six distinct TNBC subordinate types, developing tailored therapy for TNBCs is extremely difficult. Each subordinate type of breast tumor reacts to therapy differently, making BrCa extremely difficult to treat [20]. Each TNBC subtype has varied gene expressions and survival rates, as shown in Figure 1, making it more challenging to establish a precise assortment of effective and safe chemotherapeutics or medications for BrCa patients [9].

## 3. Risk Factors for Breast Cancer

BrCa remains the most common malignancy in women and the second leading cause of cancer death. Despite the urgent need for effective and novel therapies, there is considerable concern in identifying BrCa risk factors and improving chemo-preventive and lifestyle adjustment actions that can help decrease the impact of the disease. Although BRCA1 and BRCA2, as tumor-suppressor proteins, signify less than 10% of cases; their discovery has overwhelmingly influenced patient treatment [21]. Other risk factors linked with ER-positive BrCa progress, for example early menarche, early thelarche, and first pregnancy at a later age, are less well characterized and may also be linked to increased estrogen exposure [22]. Obesity and metabolic syndrome, additionally, have been recently established as significant BrCa risk factors [23,24], a link that is particularly noteworthy considering the present obesity epidemic. Even though the processes connecting BrCa and obesity are complicated, increased influence of adipokines and inflammatory cytokines, as well as increases in circulating insulin and insulin-like growth factors, local estrogen synthesis in adipose tissue, and the impact of circulating insulin and insulin-like growth factors, are all thought to play a role in disease development [25]. Surprisingly, there is much evidence relating these factors to the ER, so it is not surprising that ER-positive cancers are more common in postmenopausal women [26]. Finally, increased cholesterol, low-density lipoprotein (LDL)-cholesterol, and very-low-density lipoprotein (VLDL)-cholesterol have been identified as obesity comorbidities [26,27,28,29] and may be independent risk factors for breast cancer [30].

## 4. Mechanisms Inherent in the Clinical Actions of Cholesterol in BrCa

Several homeostatic practices are normalized in most cells to keep free cholesterol at a highly constant level: (i) splitting into the endoplasmic membranes and plasma; (ii) efflux, uptake, and de novo synthesis; and (iii) esterification via acyl-CoA: cholesterol acyltransferase (ACAT) [31]. Sterol regulatory element-binding protein-2 (SREBP2) is considered one of the most significant regulators of intracellular cholesterol levels and a transcription factor that operates as a cholesterol sensor. It is also inhibited by free cholesterol [32]. SREBP2 is kept static as a portion of a large multiprotein complex correlated with the endoplasmic reticulum. The stability of this complex is compromised when cholesterol levels fall, and SREBP2 transfers to the Golgi apparatus, where it experiences a sequence of protective handling actions that result in its activation, facilitated by the chaperone SREBP cleavage-activating protein (SCAP). Once it enters the nucleus, SREBP2 enhances the expression of genes participating in cholesterol manufacture, for instance, cholesterol import, 3-hydroxy-3-methylglutaryl-CoA reductase (HMGCR), the LDL receptor gene, and low-density lipoprotein receptor (LDLR) [32,33,34]. 

Additionally, SREBP2 LXRs play a role in preserving intracellular cholesterol homeostasis. Liver X receptor (LXRs) are ligand-controlled transcription factors positively regulated by oxysterol ligands generated in cells [35]. LXR activation restores cholesterol homeostasis in cells by upregulating the expression of cholesterol uptake genes such as the LDLR (IDOL) induction degrader process, an E3 ubiquitin ligase that causes LDLR depletion in the lysosome, and cholesterol reverse transport genes such as adenosine 5′-triphosphate (ATP)-binding cassette subfamily A1 and ATP-binding cassette subfamily G member 1 (ABCG1) [36,37]. Because of the complexity and redundancy of the pathways that maintain intracellular cholesterol homeostasis, determining how changes in circulating cholesterol can affect cancer development has been difficult. However, when there is a high need for cholesterol, such as during rapid growth, cells must disengage the systems that maintain cholesterol homeostasis. Studies of cholesterol biochemistry in immune cells called activated T lymphocytes (T cells) have shed light on how this can happen. It has been discovered that when the T cell receptor (TCR) is activated, the sulfotransferase family cytosolic 2B member 1 (SULT2B1) synthesizing, an important enzyme that has a role in sulfates and inactivates the LXR oxysterol ligands, is increased. As a result of the loss of LXR function, the cells can accumulate the cholesterol essential for new membrane synthesis [38]. It will be exciting to see if basal cell carcinoma cells utilize a similar method to accumulate the cholesterol needed for cell proliferation. In this way, the agonist-activated ER reduces LXR-mediated gene transcription in ER-positive BrCa cells, mainly affecting ABCA1 expression [39]. It will be critical to realize how cholesterol homeostasis is interrupted in the rapidly replicating BrCa cells and the processes that allow the ER and LXR to interact. While cholesterol is required for membrane formation during cell division and may be a limiting factor, it is unknown whether or not this action is harmful in and of itself. However, increased expression of the methyl transferase enhancer of zeste homolog (2EZH2) and the associated downregulation of tumor suppressors has been demonstrated to cause prostatic intraepithelial neoplasia in knockout mice without homeostatic control of cholesterol (LXR knockout mice) [40]. Because these trials used a high-fat with high cholesterol (HFHC) diet, linking the observed carcinogenic effects to cholesterol was difficult. On the other hand, increased cholesterol concentration appears to alter the biophysical properties of membranes, making lipid raft formation easier and boosting the signaling pathways process that begins at the membrane. Indeed, enhanced phosphoinositide 3-kinase (PI3K) activation and phosphorylation of AKT/protein kinase B were seen in ApoE/mice fed an HFHC diet, indicating enhanced phosphoinositide 3-kinase (PI3K) activation and phosphorylation of AKT/protein kinase B [41]. 

Furthermore, the PI3K inhibitor BKM120 (buparlisib) inhibited the formation of BrCa in these animals, implying that the PI3K/AKT signaling pathway is involved in the harmful effects of cholesterol in tumors. Given that plasma cholesterol levels in this animal model approach 2000 mg/dL, far higher than what would be considered hypercholesterolemic in humans (240 mg/dL), the significance of this finding is disputed. According to in vitro experiments, the amount of exogenous cholesterol necessary to induce cell proliferation is far lower than that required to form lipid rafts and to phosphorylate AKT [41]. 

These findings are more reliable with the assumption that cholesterol acts as a signaling molecule in cancer cells instead of the idea that cholesterol influences tumor pathophysiology by increasing lipid raft generation and membrane signaling. In this context, the cholesterol metabolite 27-hydroxycholesterol (27HC) is noticeable because it has been discovered to behave as both an endogenous ER modulator (SERM) and an LXR agonist [42,43]. 27HC acts as an ER antagonist in the cardiovascular system and an ER agonist in osteoblasts and ER-positive breast cancer [43,44,45,46,47,48]. As a result, converting cholesterol to 27HC may play a role in some of the cholesterol’s potentially harmful effects in ER-positive malignancies [39]. According to research, to affect tumor growth, cholesterol must be converted to its metabolite 27HC. This hypothesis was confirmed in the MMTVPyMT animal model. It was discovered that while a high-cholesterol diet (HCD) accelerated mammary tumor growth, it had no effect when CYP27A1, the enzyme responsible for the conversion of cholesterol to 27HC, was knocked out [39]. 

Oxysterols have also been discovered to activate the hedgehog pathway [49]. Even though the influence of oxysterols on breast cancer cells by way of this pathway has not been thoroughly investigated, cancer cells may be directly affected. While oxysterol-induced LXR activation lowers cholesterol levels in cells, reducing cancer cell production, it has been revealed that LXR activation decreases the chemokine receptor 7 (CCR7) expression in dendritic cells, diminishing its antitumor effect [50]. As a result, 27HC is expected to impact tumor biology by helping cancer cells avoid immune scrutiny and activate the ER [51].

## 5. Natural Compounds against Breast Cancer

### 5.1. Quercetin

Quercetin (QC) is a flavonol produced by plants such as *Opuntia ficus-indica*, *Lychnophora staavioides*, *Allagopappus viscosissimus*, and *Rhamnus* spp.; it belongs to the flavonoid category of polyphenols (Table 1) [52,53]. Its geographic allocation can be found in different trouser materials [54]. Various wine, plants, tea, fruits, and coffee contain these antioxidant, anticancer, and anti-inflammatory chemicals [55,56]. QC induces programmed cell death in cells from prostate, lung, breast, colon, and cervical cancers [55]. Furthermore, cancer cell proliferation is inhibited, and apoptosis is induced by nanoparticles containing QC [52]. QC lowers the representation of anti-apoptotic proteins such as Bcl-xL, survivin, and Bcl-2, while improving the representation of pro-apoptotic proteins such as Bad and Bax to promote apoptosis in cancer cells [55]. Because it affects cancer cells and causes them to undergo apoptosis, this chemical could be used to treat a variety of cancers [57]. For the prevention and treatment of BrCa, QC has shown promise [52]. QC causes apoptosis in BrCa cells, for instance, BT-20 and MCF-7 cells, by inactivating c-FLIPL and increasing DR5. QC also blocks BrCa cells (MCF-7) from proliferating by arresting the cell cycle [55]. It also suppresses the proliferation and invasiveness of BrCa stem cells (MDA-MB-231) and decreases the regulation of various proteins involved in BrCa cell growth, such as aldehyde dehydrogenase 1A1, epithelial cell adhesion proteins, etc. BrCa cell growth is inhibited by downregulated proteins, revealing the anticancer properties of QC [57]. Because QC stimulates apoptosis in BrCa cells, it can effectively manage the disease [12]. QC affects the G1 phase and causes apoptosis in MCF-7 cells by lowering cyclin D 1, P21, and Twist expression. Through the P38MAPK pathway, Qu was found to effectively reduce Twist expression. QC reduces P38MAPK phosphorylation, which is a hallmark of cell growth in MCF-7 cells [58]. This medicine reduces STAT3 signaling and suppresses HER 2 overexpression in BT-474 BC cells by activating caspase-dependent extrinsic apoptosis and increasing the sub-G0/G1 apoptotic population [59]. QC metabolites that limit MCF-7 cell development, such as QC, quercetin-3-sulfate (Q3 S), and quercetin-3-glucoside (Q3G) [60], can be listed in the order of potency Qu > Q3 S > Q3G. Q3 S and Q3G increase apoptosis and necrosis in MCF-7 cells, respectively. As a result, the anticancer activity of QC, Q3 S, and Q3G is being explored more closely as a remarkable phenomenon in cancer therapy [33]. QC has a dose-dependent inhibitory effect on cancer cell proliferation. By regulating PI3k, EGFR, and Her2/neu, QC suppresses cell proliferation [58,59,60]. Although the first anti-proliferative effect of QC is shown approximately at low doses [18], Qu inhibits cell proliferation by modulating PI3k, EGFR, and Her2/neu.

### 5.2. Tetrandrine

Tetrandrine inhibits the spread of cancer cells and possesses antiproliferative properties [61]. It is a dibenzyl tetrahydro isoquinoline alkaloid that was discovered in *Stephania tetrandra*, a medicinal Asian herb (Chinese plant) (Table 1) [61,62]. This natural molecule has been shown to induce apoptosis in prostate tumors, leukemia, melanomas, and breast tumors [61]. Tetrandrine has pharmacologic outcomes, preventing drug resistance proteins and helping to block positive ion channels [62]. This drug is used to treat a variety of cancers, affecting tumor cell resistance [63] and can reverse drug resistance in human BrCa cells [64]. Tetrandrine increases autophagy as well. Autophagic cell death thus occurs in cells tolerant to apoptosis and, as a result, to cell death in general [65]. Tetrandrine is a promising medicine for the treatment of various malignancies because it stops cancer cells from multiplying. Tetrandrine kills inflammatory and tumor-initiating cells in the breast, preventing them from multiplying. This medicine reduces the creation of mammospheres, a marker of cancer cell proliferation, and aldehyde dehydrogenase protein expression. In SUM-149 and SUM-159 BrCa cells, tetrandrine exhibits an antiproliferative impact. Furthermore, because aldehyde dehydrogenase proteins are connected to BrCa cell growth, downregulating them has antiproliferative effects [62,66]. In BrCa MCF-7/TAM cells, tetrandrine overturns tamoxifen resistance. As an autophagy activator, tetrandrine induces autophagy in numerous BrCa cell lines and accelerates cell death in cells resistant to apoptosis. Apoptosis-resistant cell types have lower expression of caspase 3, caspase 7, and Bax. As a result, tetrandrine effectively induces cell death in cancer cells [65], and has characteristics that make it a promising therapy for the inhibition and therapy of BrCa [64].

As a checkpoint inhibitor of the cell cycle in cancer cells, tetrandrine has been shown to be able to halt cell proliferation, followed by apoptosis, either by the activation of caspase or the FASL-mediated route. Tetrandrine suppresses CDK4 and CDK2-CycE in the colon, endothelium, and hepatocellular carcinomas by inhibiting the ATP binding site of CDKs, similar to the soybean isoflavone daidzein metabolite 3′,4′,7-trihydroxyisoflavone and preventing the G1–S transition in cells [67]. Aside from CDK1, CDK2, and CDK6, tetrandrine has also been found to have no effect on these enzymes at the pharmacological concentrations. The downregulation of hyperphosphorylated RB by tetrandrine may also contribute to the repression of CDK4, CDK6, and CycD1 concentrations, which otherwise assist in the transition from G1 to S. P53 and CIP/KIP family proteins were also elevated by tetrandrine in a time-dependent manner in several malignancies [67,68], and the cells were prevented from entering the G1 phase [66,67,68]. This shows that tetrandrine could be used as a CDK inhibitor. In addition to its CDK inhibitory effect, tetrandrine may also boost the proteolysis of the main regulator of cell cycle CycD1 by activating GSK 3, comparable to resveratrol, cycloheximide, and aspirin [69]. The PI3K/AKT/mTOR pathway, which is essential for cell survival, proliferation, migration, and angiogenesis in mouse endothelial cells, was likewise inhibited by tetrandrine, resulting in cell cycle arrest [67,68,69].

### 5.3. Thymoquinone

*Nigella sativa* seeds, grown in Mediterranean and Western Asian countries, contain thymoquinone (TQ) (Table 1) [70,71]. This chemical has been proven to be resistant to myeloblastic pancreatic adenocarcinoma, leukemia, osteosarcoma, breast cancer, liver malignancies, ovarian cancer, and colorectal malignancies [70,71,72,73,74]. Several proteins, including p53, p73, STAT3, nuclear factor kappa-B (NF-κB), PPAR, and reactive oxygen species (ROS), are involved in the anticancer effect of TQ [74]. TQ increases pro-apoptotic protein levels while decreasing anti-apoptotic protein levels in MCF-7, HCT-116, and HL-60 cancer cells, implying antiproliferative characteristics [72,75]. As a result, TQ can be used as a cancer therapy. TQ has anticancer effects in BrCa cells. Boosting p38 phosphorylation and ROS signaling inhibits migration and increases death in BrCa cells. The antiproliferative characteristics of TW are demonstrated by its ability to block the synthesis of anti-apoptotic proteins such as survivin, Bcl-xL, and Bcl-2 [76]. TQ is a successful treatment for BrCa, as seen by decreased anti-apoptotic proteins, increased p38 phosphorylation, and decreasing breast tumors [74]. 

In addition to its potential to reduce cell production, TQ inhibits S phase molecules and causes sub-G1 arrest in cells. TQ induces apoptosis via regulating several targets, both p53 dependent and p53 independent. TQ stops cell proliferation and tumor growth by altering the cell cycle and targeting NF-κB. Even low concentrations of BrCa cells are blocked after long-term therapy [76]. TQ causes poly (ADP-ribose) polymerase to break, H2AX to increase, Akt phosphorylation to decrease, and a reduction in X-linked inhibitor of apoptosis [77]. It also acts as a PPAR ligand, inhibiting the growth of BrCa MCF-7/DOX cells [78]. TQ decreases Akt phosphorylation while increasing PTEN protein presence. TQ prevents cells from growth by inhibiting Akt phosphorylation, which is required for cell survival. During the G2/M phase, TQ inhibits MCF-7/DOX cells [79,80]. TQ inhibits Akt via lowering cyclin D1 and cyclin E production, as well as the phosphorylation of 4E-BP1, eIF4E, S6R, and p70S6K [80]. As a result, TQ is an efficient treatment for the condition because it causes apoptosis in BrCa cells [12].

**Table 1:** The efficacy of natural compounds in the treatment of BrCa.

Natural Compound and Class	Chemical Formulas	Source	Mechanism of Action	References
Quercetin (flavonoid)	C_15_H_10_O_7_	*Allagopappus viscosissimus*, *Opuntia ficus-indica var. saboten*, *Lychnophora staavioides*, and *Rhamnus species*	Programmed cell death and the cell cycle are promoted, and breast cancer stem cells (BCSCs) are kept from invasion	[45,53,55,81,82,83,84]
Tetrandrine (alkaloid)	C_38_H_42_N_2_O_6_	*Stephania tetrandra*	Blocks positive ion channels, overcomes drug resistance, boosts autophagy, and triggers cell death	[61,62,65]
Thymoquinone (4-benzoquinone)	C_10_H_12_O_2_	*Nigella Sativa*	Both p53-dependent and p53-independent mechanisms increase apoptosis; cell cycle arrest triggers p38 and ROS signaling; NF-κB is a tumor-suppressing protein. The peroxisome proliferator-activated receptor (PPAR) activation pathway has improved, as has PPAR activity; phosphorylation of Akt, 4E-BP1, eIF4E, S6R, and p70S6K has decreased	[70,72,74,76,78,80]
Resveratrol (phytoalexin)	C_14_H_12_O_3_	*Polygonum cuspidatum*	Encourages cell cycle arrest and death; prevents carcinogenesis, DNA damage, and cancer spread; Cells genetic and epigenetic profiles are altered, and COX activity is inhibited; NF-κB DNA’s binding activity is reduced, and cell viability, glucose ingesting, and ATP content are all reduced; TGFβ1 expression is suppressed; BCSC survival is reduced; Wnt/β-catenin signaling pathway is inhibited, resulting in autophagy; signaling between PI3K, Akt, and mTOR is suppressed	[85,86,87,88,89,90,91,92]
Honokiol (neolignan biphenols)	C_18_H_18_O_2_	*Magnolia grandiflora*	Autophosphorylation inhibits angiogenesis, tumor cell proliferation, and programmed cell death; the PI3K/mTOR pathway governs immune resistance; inhibits angiogenesis, tumor cell proliferation, and death; suppresses Wnt1-MTA1-β-catenin signaling induced by leptin; STAT3 phosphorylation is reduced, and phospholipase D (PLD) activity is inhibited; induces cell cycle arrest and decreases mammosphere development, aldehyde dehydrogenases (ALDH) activity, and expression of iPSC inducers; EGFR is inhibited, and c-Src phosphorylation is suppressed	[93,94,95,96,97,98]
Garcinol (polyisoprenylated benzophenone)	C_38_H_50_O_6_,	*Garcinia indica*	Regulates the NF-κB signaling pathway; reduces histone acetyltransferases and ROS; induces cell cycle arrest; reverses EMT markers, and governs the β-catenin and Wnt signaling pathways	[99,100,101,102]
Biochanin A (flavonoid)	C_16_H_12_O_5_	*Trifolium pratense*	Biochanin A inhibited the aromatase enzyme activity and prevented cell proliferation in MCF-7 cells that had been stably transfected with the CYP19 gene. Biochanin A was reported to reduce aromatase enzyme activity and mRNA expression in SK-BR3 cells (ER-negative BrCa cells)	[103,104,105]
Lycopene (tetraterpenoids)	C_40_H_56_	Tomatoes, carrots, watermelon, papaya, and cherries all contain lycopene, a vivid red carotene pigment that belongs to the tetra terpenoids family	BrCa cells regulate several genes involved in DNA repair, cell cycle control, and apoptosis, making them potent antioxidants	[106,107,108]
Shikonin (hydroxy-1,4-naphthoquinone)	C_16_H_16_O_5_	Lithospermum erythrorhizon’s root extract	Shikonin inhibits estrogen-encouraged cell production and initiates ER ubiquitination, promoting ER breakdown in ER-positive breast cells. It induces necroptosis-like death in ER-positive BrCa cells	[109,110]
Sulforaphane (isothiocyanate)	C_6_H_11_NOS_2_	Broccoli, water lily, broccoli sprouts, cabbage, and kale	In BrCa cells, sulforaphane has been shown to prevent tubulin polymerization. It can cause both cell cycle detention and apoptosis in BrCa cells	[111,112]
Caffeic acid (phenolic compound)	C_9_H_8_O_4_	*Echinacea purpurea*	Echinacea includes flavonoids, which stimulate the immune system. It boosts lymphocyte activity, which encourages macrophage phagocytosis and natural killer cell activity, triggering interferon assembly and minimizing the adverse effects of chemotherapy and radiation therapy. It also helps people extend their life expectancy as their cancer advances. Echinacea juice in commercial formulations has been demonstrated to increase macrophage cytokine production. The activation and proliferation of T-cells and B-cells has fewer apparent implications. Several components of echinacea have been shown to contribute to the immune system’s unique effects	[82,113]
Alliin, and Allicin (sulfoxide)	C6H_11_NO_3_S, C_6_H_10_OS_2_	*Allium sativum*	Garlic’s anticancer benefits come from its high organic sulfides and polysulfides composition. The mechanisms of antitumor activity activating lymphocytes and macrophages are the destruction of malignant cells and interfering with tumor cell metabolism	[114]
Curcumin (flavonoid)	C_21_H_20_O_6_	*Curcuma longa*	*Curcuma longa* (turmeric) gives food a dark yellow color. The active element in turmeric, curcumin, can be found in the rhizome and rootstock. Curcumin’s phenolic compounds have been demonstrated to have anticancer properties. Turmeric protects against lung, breast, skin, and stomach cancers	[114,115,116,117]
Luteolin (flavonoid)	C_15_H_10_O_6_	*Arctium lappa*	Antioxidants of the flavonoid and polyphenol are found in burdock root, suppressing tumor growth. Root extract protects normal physiological cells from toxic substances and helps to prevent cell mutations. The most crucial active element in burdock is tannin, a phenolic substance. It activates macrophages, inhibits cancer spread, and maintains immune-modulatory capacities	[118,119]
Carotenoids (Tetraterpenoids)	C_40_H_64_	*Rosehips*	Carotenoids are potent antioxidants with therapeutic properties, such as scavenging free radicals, protecting cells from oxidative stress, illuminating gap intersections, stimulating the immune system, and regulating enzyme activity, all of which contribute to cancer production and boost the body’s immune system activity	[120]
Epigallocatechin gallate (catechin)	C_22_H_18_O_11_	*Camellia sinensis*	Green tea possesses cancer-fighting and antimutagenic properties. EGCG protects cells against DNA damage caused by reactive oxygen species. Green tea polyphenols, according to animal studies, inhibit cancer cell division and cause tumor cell necrosis and death	[121,122,123,124]

### 5.4. Resveratrol

Resveratrol (trans-3,5,4′-trihydroxystilbene, RES) is a polyphenolic molecule found in grapes, peanuts, soybeans, pomegranates, and berries (Table 1) [125,126]. Polygonum cuspidatum, sometimes known as Japanese knotweed, is an anti-inflammatory herb that contains a lot of resveratrol. This plant has been utilized in Asian countries to prevent and treat various diseases, including cancer, for hundreds of years. RES kills tumor cells because it arrests the cell cycle and causes death. In addition to inhibiting tumor-derived nitric oxide synthase, RES can reduce DNA damage and delay tumor growth by acting as an antioxidant [85]. This chemical also alters genetic and epigenetic characteristics of tumor cells, indicating anticancer activity [86]. It also reduces the ability of NF-κB to bind to DNA. RES inhibits tumor growth by lowering the binding activity of this factor, which increases the transcription of genes that promote tumor cell proliferation [85,127,128]. RES decreases intracellular ROS, mitochondrial membrane potential, mTOR, RP-S6, and 4EBP1 phosphorylation. By inhibiting viruses, it prevents inflammation and leukemia. RES has been shown to have apoptosis-inducing and neuroprotective effects [87]. A variety of methods, including targeting p53, Rb, and cell cycle kinases, are used by RES to cause apoptosis [88]. RES destroys cancer cell production and induces programmed cell death [129]. It is used to treat colorectal, liver, pancreas, prostate, and breast malignancies [85,130]. Because of its multi-target efficiency, suitability, and cost-efficacy, RES is an efficient therapy for various cancers [131]. 

RES in the triple-negative BrCa cell lines MDA-MB-231 and MDA-MB-231/PacR, RES reduces cell growth, stimulates aging, lowers survivin construction, and stimulates apoptosis. It induces apoptosis by stimulating caspase 7 [129]. In MCF-7 cells, RES suppresses phosphofructokinase (PFK), and reduces glucose consumption, cell viability, and ATP content. In this way, RES prevents these cells from surviving and multiplying [89]. RES suppresses tumor growth in a variety of animal models. In female rats, for example, it diminishes tumor development. In rats, RES destroys COX2 production and attaches NF-κB to DNA. It decreases the representation of 5-LOX, TGF1, and NF-κB, inhibits single-strand DNA representation, and lowers DNA damage. RES also prevents BrCa cancers from forming and spreading [85]. RES controls apoptosis in BrCa cells via modulating tumor-suppressive miRNAs such as miR-122-5p, miR-200c-3p, miR-409-3p, miR-125b-5p, and miR-542-3p. In MCF-7 cells, miR-542-3p prevents apoptosis, whereas in MDA-MB-231 cells, miR-122-5p inhibits apoptosis [90]. 

In MCF-7 cells, RES also boosts the synthesis of ASPP1, a p53 protein activator that stimulates apoptosis. RES also enhances the representation of BAX and p21 [132]. It inhibits the progression of cancer by inhibiting Bcl-2. Caspases 7 and 9 are stimulated by RES, which also increases p53 expression and decreases procaspase 8. Furthermore, cell cycle arrest encouraged by RES in the S phase raises p-Chk2 levels. RES prevents CDK7 activity, and the active form of CDK2 is reduced [91]. Through plasma membrane integrin v3, it promotes p53-dependent apoptosis, showing an anti-proliferative action [91]. By decreasing FASN, RES alters cell cycle progression and causes apoptosis in HER2-positive BrCa cells [92]. Thus, RES could play a role as possible BrCa therapy.

### 5.5. Honokiol

Honokiol (HNK), an organic chemical originating from *Magnolia grandiflora* from the Southeast United States and other world territories (Table 1) [133], retains antibacterial, antioxidant, and anti-inflammatory properties [93,94]. It prevents angiogenesis, combined with tumor propagate [93], and vascular endothelial growth, with antitumor effects [134]. It lowers tumor xenograft development in mice and suppresses cell proliferation in vitro. HNK induces caspase-dependent apoptosis in B-cell chronic lymphocytic leukemia cells [93]. This is achieved by mechanisms that are not p53-dependent [94]. Prostate cancer cells are also prevented from spreading to the bones by HNK [93]. Glioma, breast, and prostate cancer cells also lower immunoresistance mediated by the PI3K/mTOR pathway [95]. 

HNK also differentiates human HL-60 cells. In HUVECs and mice, the drug suppresses vascular endothelial growth factor (VEGF)-induced kinase insert domain receptor (KDR) autophosphorylation and angiosarcoma development. It also inhibits the growth of RKO colon cancer cells and RKO solid tumors in mice. HNK helps prolog survival in mice with solid tumors [94]. Consequently, HNK has the potential to be used to treat cancer. HNK may be useful in the treatment of BrCa. The substance reduces BrCa cell multiplication while enhancing the function of other drugs used for treatment. In BrCa cells from mice, HNK causes cell cycle arrest [135,136]. In MDA-MB-231 cells, it improves caspase 3 activation and stimulates pro-apoptotic features. In HNK-exposed mice, tumor cell production is inhibited [137]. Wnt1–MTA1–catenin signaling is also inhibited by HNK, which is facilitated by leptin. Releasing repressor-STAT3 suppresses STAT3 phosphorylation and activates miRNAs [96]. Cancer cells have a better chance of surviving when their PLD activity is boosted. As a result, a medication that blocks PLD activity should effectively reduce BrCa cell proliferation. HNK inhibits PLD activity, preventing cancer cells from developing. Increased PLD activity correlates with Ras activation in MDA-MB-231 cells, and HNK reduces both PLD activity and Ras activation. HNK has potential as a BrCa therapeutic drug since it decreases Ras and PLD activity, which enhances cell survival [93]. 

Additionally, in BrCa cells, HNK inhibits mammosphere development, ALDH activity, and the production of iPSC inducers, indicating that it may have anticancer properties. LKB1 promotes HNK, which lowers the stem-like phenotype of BrCa cells by inactivating STAT3 and downregulating iPSC inducers [94]. It also stops the proliferation of MDA-MB-231 BrCa cells in the G0/G1 phase because of HNK. In addition to the rise in p27Kip-1 (a cyclin-dependent kinase (CDK) inhibitor), CDK4, cyclin D1, CDK2, cyclin A, and cyclin E levels are increased. As a result of the activation of a caspase cascade by HNK, PARP cleavage and DNA fragmentation are also increased. Because of this, the apoptosis rate of the brca-expressing cells rises sharply. As a result, HNK inhibits cell development by altering the signaling pathways in the cells themselves. Inhibition of the receptor tyrosine kinase, EGFR, which raises EGFR levels by phosphorylating it at Tyr845, is the effect of this drug. Phosphorylated c-Src protein is prevented from being activated by HNK. The proliferative activities of HNK are connected to inhibiting the components of BrCa cell proliferation and angiogenesis in MDA-MB-231 cells. Additionally, HNK reduces Akt and c-Src expression, resulting in increased cell survival and decreased apoptosis. Akt and c-Src inhibition are regulated by Hsp90, while HNK inhibits Hsp90. C-Src and Akt production is reduced by HNK, which makes it an excellent treatment option for BrCa [98].

### 5.6. Garcinol

Garcinol is a polyisoprenylated benzophenone from the Garcinia plant that is prized in India, Africa, and China (Table 1) [138]. This acetyltransferase inhibitor is found in tropical plants [139]. In addition to its antioxidant capabilities, garcinol is increasingly employed for its anticancer potential. Garcinol reduces ROS and inhibits histone acetyltransferases [101]. Consequently, it appears to be a viable cancer treatment. In BrCa cells, altering the NF-κB signaling pathway inhibits cell development and causes apoptosis. Garcinol inhibits E2-induced production and promotes apoptosis in MCF-7 BrCa cells and also inhibits the formation of ac-H3, ac-H4, and NF-κB/ac-p65 in these cells. It also inhibits the nuclear translocation of NF-κB/p65 and the mRNA and protein expression of cyclin D1, Bcl-2, and Bcl-xL. Garcinol suppresses MCF-7 BrCa cell formation in the NF-κB pathway by altering gene expression and reducing ac-p65 expression [139]. 

Garcinol affects EMT markers in MDA-MB-231 and BT-549 BrCa cells. The expression of miRNAs from the miR-200 and let-7 families is boosted by garcinol. It also boosts catenin phosphorylation while lowering its nuclear localization. Garcinol reduces cancer cell invasion by inhibiting the Wnt signaling pathway. In mice, garcinol lowers the levels of NF-κB, miRNAs, vimentin, and nuclear catenin. As a result, the anticarcinogenic effects of garcinol on BrCa are linked to the reversal of the EMT phenotype [98]. Garcinol inhibits the NF-κB signaling pathway, which slows apoptosis. Garcinol reduces constitutive NF-kappa events in MCF-7 and MDA-MB-231 BrCa cells, which coincides with the downregulation of genes associated with NF-κB. This substance also slows the growth of BrCa and reduces apoptosis. Garcinol reduces the representation of 9-nAChR and cyclin D3, two proteins implicated in nicotine-induced BrCa, in MDA-MB-231 BrCa cells, inhibiting cell migration. Garcinol is a cancer-fighting drug [131] that may be used to treat BrCa by preventing the mechanisms that cause disease progression [102].

### 5.7. Biochanin A

Biochanin A, an isoflavone with anticancer properties, is found in red clover (*Trifolium pratense*) (Table 1) [140]. According to Wang et al. [103], biochanin A inhibited aromatase enzyme activity and stopped cell proliferation in MCF-7 cells that were stably transfected with the CYP19 gene. Biochanin A decreased aromatase enzyme activity and mRNA expression in SK-BR3 cells. Biochanin A is a metabolite that has been proven to serve as an AI by inhibiting the action of the agents I.3/II. Biochanin A is better tolerated than genistein in the human mammary epithelial cells (HMECs), MCF 12A, and MCF-7 cells and results in a positive expression of tumor suppressor genes [104,105]. Young et al. [141] made a similar observation, finding that biochanin A stimulated tumor suppressor gene expression more effectively than genistein. According to Bhushan et al. [104], in SK-BR-3 cancer cells, biochanin A lowered cell survival, signaling pathways, and invasive enzyme expression and activity. Moon et al. [142], reported that biochanin A at doses of 5 or 15 mg/kg per day successfully reduced the growth of estrogen-dependent MCF-7 tumors in a xenograft mice model. The role of biochanin A role in other routes of breast cancer progression is unknown, and more clinical trial research is needed to better understand its bioavailability, treatment regimen, and metabolic profile in different types of breast cancer. Future studies should focus on the effects of biochanin A in ER-negative or triple-negative breast cancer cells because most research has been performed in ER-positive cell lines [13].

Biochanin A suppresses the proliferation of cancer cells in SK-Mel-28 melanoma cells in a dose and time-dependent manner. Biochanin A also caused these cells to glow orange/red, and the intensity of this red fluorescence increased when the drug dose was raised, demonstrating that biochanin A causes apoptosis in SK-Mel-28 melanoma cells [143]. The effect of biochanin A (20 M and 70 M) on the ability of temozolomide to reduce the number of colonies formed, as evaluated by the colony formation assay, is enhanced. We also looked at how temozolomide (100 M) affected cell signaling, invasive processes, and transcription factors alone and in conjunction with biochanin A (20 M and 70 M). The protein expression of p-EGFR, p-ERK, uPAR, and MMP-2 was decreased, according to Western blot examination. Our findings suggest that using biochanin A with temozolomide can improve the anticancer effect of temozolomide on glioblastoma cells [144]. These findings aid in our understanding of how biochanin A can be used to develop new, more effective combination therapies. Further research will be conducted to determine the mechanism(s) by which this combination prevents U-87 MG cells from invading.

### 5.8. Lycopene

Lycopene, a vivid red carotene pigment found in tomatoes, carrots, watermelon, papaya, and cherries, belongs to the tetraterpenoids family (Table 1). It controls several genes in breast cancer cells involved in DNA repair, cell cycle control, and apoptosis [107,108]. Research by King-Batoon et al. [145] showed that lycopene altered the GSTP1 gene in BrCa cells. With the MDA-MB-468 cell line, lycopene (2 µM for one week) boosted GSTP1 expression and demethylated the GSTP1 promoter; however, this was not the case in MCF-7 BrCa cells. In MCF-7 and MDA-MB-468 BrCa cells, lycopene treatment did not affect the expression of other genes such as RAR2 and HIN1 [145,146]. 

By changing cell growth factor signaling pathways and inducing cell cycle arrest, lycopene can cause cell death and anticancer effects. Takeshima et al. [147] conducted a similar experiment and found that lycopene suppressed triple-negative breast cancer cell proliferation by preventing Akt activation via its downstream route and upregulating proapoptotic Bax without impacting antiapoptotic Bcl-xL. Lycopene has also been reported to lower cyclin D1 levels via upregulating p21 and maintaining ERK1/2 activation. Peng et al. [148] discovered the antiproliferative mechanism of lycopene in MCF-7 cells, where it reduced cell proliferation and increased apoptosis through regulating the expression of p53 and Bax. The mechanisms of lycopene in ER subtypes are still under discussion, with one study finding a negative association between lycopene and other carotenoids and ER subtypes [149], and another group demonstrating that consuming carotenoids like lycopene can reduce the risk of ER subtypes [150]. Hence, more research is needed to understand how lycopene functions as a chemopreventive agent in different pathways and breast cancer subtypes. Additionally, Rao and Shen [151] recommended taking 5 to 10 mg of lycopene each day to minimize free radical damage to cells. In prostate cancer patients, supplementing dietary extracts with lycopene lowered oxidative stress and carcinogenesis [152]. Few studies on the health advantages of lycopene alone have been conducted, and further clinical trial-based studies examining the chemopreventive action of lycopene are required [13].

### 5.9. Shikonin

Shikonin, a chemical obtained primarily from *Lithospermum erythrorhizon*’s root extract, has been demonstrated to have anticancer, anti-inflammatory, wound-healing, antiviral, and other biological characteristics (Table 1) [153]. Shikonin inhibits estrogen-stimulated cell proliferation and promotes ER breakdown in ER-positive breast cells by initiating ER ubiquitination and inhibiting cell proliferation [109]. Shikonin suppressed pS2, c-myc, and estrogen-sensitive gene promoters in BrCa cells and protected against estrogen-induced DNA damage via activating the Nrf2 pathway, according to the Yao [154] group. Shikonin inhibits the expression of steroid sulfatase genes while inducing necroptosis-like death in ER-positive BrCa cells [110,155]. Shikonin targets several pathways during apoptosis stimulation, including caspase-3 activation, NF-κB pathway inhibition, and alteration of apoptosis-related genes Bcl-2 and Bax. Shikonin blocks the NF-κB path by reducing IB-phosphorylation and suppressing p65 [156]. Shikonin reduced BrCa cell migration and invasion by altering matrix metalloproteinase-9 (MMP-9) [157]. 

Wang et al. [158], on the other hand, discovered that shikonin reduced the proliferation of ER-negative human breast cancer cells via reducing HIF-1a expression. Shikonin also improves taxol chemosensitivity in ER-negative human breast cells, causing cell cycle arrest in the G2/M phase, and inhibits ERK, Akt, and p70S6 kinase activation, all of which are important in cancer treatment resistance [159]. According to Zhang et al. [160], shikonin suppresses tamoxifen resistance in MCF-7R BrCa cells by activating uc.57 and inhibiting the PI3K/Akt and MAPK signaling pathways downregulating BCL11A. Shikonin has been proven to be less dangerous in vivo pharmacokinetics studies [161], and it has the potential to be examined further in breast cancer therapy trials. More research is needed to enhance the bioavailability profile of shikonin and undergoes significant first-pass metabolism [162]. Furthermore, further preclinical evidence is required for the clinical use of shikonin. To better assess the efficacy of shikonin in single and adjuvant breast cancer therapy, clinical trials are required [13]. 

### 5.10. Sulforaphane

Sulforaphane (SFN) is an isothiocyanate found in broccoli, water lily, broccoli sprouts, cabbage, and kale (Table 1) [163] that has been demonstrated to inhibit proliferation, angiogenesis, and metastasis in cancer cells. It can cause both cell cycle arrest and apoptosis in BrCa cells. Bishayee [111] discovered that SFN treatment inhibited hTERT (human telomerase reverse transcriptase) in MCF-7 and MDA-MB-231 breast cancer cells via an epigenetic mechanism involving DNA methylation and histone changes in MCF-7 and MDA-MB-231 breast cancer cells in a dose- and time-dependent manner via an epigenetic mechanism involving DNA methylation and histone changes in MCF-7 and MDA-MB-231 cells. SFN has been demonstrated to have chemopreventive effects in human breast cancer cell lines through increasing cyclin B1 expression and activating the poly(ADP-ribose) polymerase one and caspase family proteins, resulting in G2/M phase cell cycle arrest and apoptosis [164,165]. SFN has been shown to inhibit tubulin polymerization [112]. The nuclear factor kappa B signaling pathway was downregulated after SFN treatment in cancer cell lines. According to Kim et al. [166,167], SFN phosphorylated Akt serine/threonine kinase and reduced Bcl-2 expression. According to one study, SFN boosts paclitaxel chemosensitivity in BrCa cells. SFN also affects hTERT and ER gene expression, causing epigenetic changes [168,169]. 

According to Li et al. [168], SFN improved tamoxifen sensitivity via epigenetic reactivation of ER in ER-negative BrCa, according to researchers who conducted the studies, both in vitro and in vivo. In another study [170], SFN lowers the synthesis of NF-κB and COX-2 by inhibiting signaling pathways mediated by ERK1/2-IKK and NAK-IKK. During their investigation, Li et al. [171] discovered that SFN therapy reduced the quantity and size of mammospheres and the ALDH+ cell population in human BrCa cell lines. Daily sulforaphane therapy at 50 mg/kg for two weeks reduced ALDH+ and downregulated the Wnt/β-catenin self-renewal pathways in the NOD/SCID xenograft model. SFN reduced SOX9 and ALDH1 expression in an ER-negative/basal-like DCIS model, leading to tumor elimination in vivo, according to a separate study by the Li group [172]. This group [172] also found that SFN caused significant changes in exosomal secretion in DCIS stem-like cells, causing them to become more like non-stem cancer cells [173]. SFN has the power to reprogram and destroy cancer stem cells (CSCs), according to these investigations [174]. 

SFN has a dose-dependent chemopreventive effect in human breast tissue, according to clinical investigations [102]. Despite the fact that SFN is well-tolerated, has no discernible toxicity in humans, and may reach beneficial levels in plasma and tissue, its absolute bioavailability decreased when the dosage was raised [166]. As a consequence, sulforaphane might be a valuable adjunct to chemotherapeutic treatments, particularly because most drugs fail to eliminate CSCs, which can lead to tumor resistance and recurrence [167]. More studies, especially large population-based studies, are needed to ensure the treatment and effectiveness of SFN on chemopreventive modulation. 

### 5.11. Echinacea

Echinacea belongs to the Asteraceae family. It is an aromatic plant endemic to the Great Plains, eastern North America, and Europe. *Echinacea purpurea*, *Echinacea angustifolia*, and *Echinacea pallida* are the three most popular species utilized in herbal remedies. On the other hand, *E. purpurea* is the most often employed species in research and treatment of various ailments such as respiratory infections, skin infections, allergic conditions, and so on. Purple coneflower, Kansas snakeroot, and black Sampson are all names for Echinacea. According to researchers, *E. purpurea* increases the number of natural killer cells in the mice tested. In the future, *E. purpurea* could be used as an anticancer medication (Table 1) [175]. Flavonoids in echinacea help to boost the immune system. According to Winston et al. [113], flavonoids increase lymphocyte activity, facilitating macrophage phagocytosis and natural killer cell activity, triggering interferon assembly and reducing the adverse effects of radiation and chemotherapy. It also aids extends survival in patients with disease progression. Echinacea juice has been shown to boost macrophage cytokine production in commercial formulations. T cell and B cell activation and proliferation appear to have fewer consequences. Echinacea’s chemical compounds have been shown to contribute to the distinct changes in the immune system [113].

### 5.12. Garlic

For hundreds of years, garlic (*Allium sativum*) has been used to treat various diseases. There are hundreds of therapeutically helpful secondary metabolites involved, including alliin, alliinase, and allicin, to name a few. Alliin, an amino acid transformed to allicin after crumpled rhizomes, is found in garlic oil. Allicin is a sulfur-containing molecule responsible for the garlic odor and its medicinal properties (Table 1). Another sulfur-binding molecule is ajoene, a sulfur-binding component found in garlic oil [176,177]. The anticancer benefits of garlic come from its high organic content of sulfides and polysulfides. The mechanisms of antitumor activity is through activating lymphocytes and macrophages, causing the destruction of malignant cells and interfering with tumor cell metabolism [114]. According to studies, garlic boosts the number of suppressor T cells and converts lymphocytes into the type that kills cancer cells. Changes in the adhesion and attachment of malignant cells circulating through blood vessels are employed to prevent metastases. By strengthening the immune system, increasing the clearance of carcinogens from the body, and enhancing the activity of detoxification enzymes, ripe garlic extract protects DNA from carcinogens. Researchers have discovered that ripened garlic extract can help prevent cancers from spreading to the colon, stomach, breast, lungs, and bladder. Garlic extract has been demonstrated to minimize chemotherapy and radiotherapy adverse effects so they can be used as adjacent therapy with other anticancer agents [114,178]. 

### 5.13. Turmeric

*Curcuma longa* is the scientific name for turmeric. Turmeric gives food a dark yellow color (Table 1). The active element in turmeric is curcumin, which is found in the rhizome and rootstock. The phenolic compounds of curcumin have been demonstrated to have anticancer properties. Turmeric inhibits lung, breast, skin, and stomach cancers [114]. Curcumin, an antioxidant, affects the formation of eicosanoids such as prostaglandin E-2 (PGE-2). In humans, it also possesses anti-inflammatory properties. Curcumin has been shown to prevent cancer growth at all stages, including initiation, promotion, and propagation. Turmeric inhibits the development of nitrosamine, which boosts the natural antioxidant activity of the body. Curcumin increases glutathione and other non-protein sulfhydryls in the body, interacting directly with various enzymes [178]. Furthermore, more preclinical evidence is needed before turmeric may be used in clinical settings. Clinical studies are needed to properly analyze the effectiveness of turmeric in single and adjuvant breast cancer treatment.

### 5.14. Burdock

*Arctium lappa* is the scientific name for burdock. Its roots can be found and utilized all over Europe and Asia (Table 1). In herbal medicine, burdock is used to cure a variety of ailments. It has a gummy texture and a sweet flavor. Burdock was initially used to treat arthritis, tonsillitis, and measles; its anticancer properties were discovered later. The active components influence oncogene alterations. It reduces pain, shrinks tumors, and improves life expectancy. To keep cancer cells growing and dividing rapidly, they need a lot of resources.

Cancer cells have a high stress tolerance and may thrive under harsh conditions such as low oxygen and low carbohydrates. The active substance in burdock seeds is arctigenin. Arctigenin may kill malignant cells even when nutrients are scarce [172]. Burdock root contains flavonoid and polyphenol antioxidants that inhibit tumor development. The root extract helps to prevent cell mutations and protects normal physiological cells from hazardous substances. The most important active ingredient in burdock is tannin, a phenolic substance. It stimulates macrophages, inhibits cancer spread, and maintains immune-modulatory functions [173].

### 5.15. Carotenoids

Rose hips (Table 1) are a green plant with a leaf that contains an active ingredient known as carotenoids. Aromatic herbs used as coloring agents include saffron, annatto, and paprika. The consumption of fruits and vegetables has been linked to reducing tumor growth in numerous ways. Carotenoids in the diet also reduce the chance of tumor growth [179]. They are potent antioxidants with a wide range of medicinal characteristics that help prevent cancer and boost the immune system’s performance [120,123]. However, before carotenoids may be employed in therapeutic settings, further preclinical evidence is required. Clinical trials are required to thoroughly assess the efficacy of carotenoids in the treatment of single and adjuvant breast cancer.

### 5.16. Green Tea

*Camellia sinensis* is the scientific name for green tea. Polyphenolic substances are thought to have anticancer properties. *C. sinensis* contains modest amounts of epigallocatechin gallate (EGCG), a polyphenol (Table 1). Green tea has been proven to have anticancer and antimutagenic properties in studies. EGCG protects cells from DNA damage caused by reactive oxygen species [123]. Green tea polyphenols, according to animal studies, inhibit cancer cell proliferation and cause tumor cell necrosis and death [124]. Tea catechins improve immunity and prevent tumor cells from spreading and developing new blood vessels. Tea and its primary catechins can help prevent cancer in various organs. Green tea can help to reduce the harmful effects of radiation. The antioxidant activities in tea are responsible for its health advantages [180].

## 6. Combination Effects

**Figure 2:**
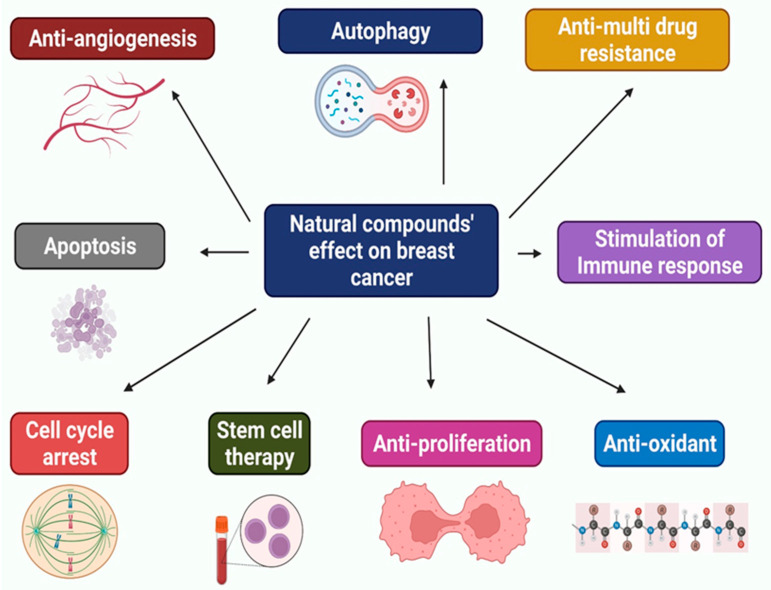
Multifunctional effects of natural compounds on breast cancer.

In the treatment of breast cancer, the combination of drugs is progressively developing. The combination therapy should result in a shorter treatment schedule, fewer side effects, and reduced expenses. Furthermore, combining therapy can improve BrCa patients’ quality of life. When used in conjunction with chemotherapeutics, natural substances and herbal medicine have been found to boost therapeutic efficacy, minimize toxicity, and inhibit resistance to various drugs [181]. Table 2 indicates the mode of action of a natural chemical mixed with other natural substances or therapeutic agents in BrCa therapy, showing effectiveness at low concentrations of carcinogenic materials. These compounds could be employed as BrCa treatments (Figure 2). This combined therapy approach is a revolutionary therapeutic option since it is more effective than single therapies. Cancer cells can by killed by combination therapies, altering the tumor environment and immune response [10]. Combination therapy is expected to reveal the benefits of several methods for suppressing cancer cells. This concept demonstrates the evolution of cancer therapy over time. Improvements in cancer treatment will provide patients with more effective treatment options [182].

## 7. Future Prospect of Herbal Management

Many roadblocks are presently obstructing traditional breast cancer therapy choices, the most notable is the dangerous side effects of drug resistance. Chemotherapy and radiotherapy have many unavoidable side effects in patients. The response to these drugs has once again decreased due to drug resistance. Natural compounds obtained from food can help in this situation since they can work with many chemotherapeutics to boost their potency. A range of natural compounds has been found to have a perfect effect in treating breast cancer when coupled with other medications. This method has been used to document various pharmaceutical and food component combinations. A synergistic effect of genistein and doxorubicin [191], equol induces tamoxifen efficacy; Pomegranate has also been studied for its capacity to enhance cell death and reduce tamoxifen-induced cell viability inhibition [192]. 3,3′-Diindolylmethane (DIM) works in conjunction with paclitaxel to promote apoptosis [193]. All anti-BrCa drugs, including tamoxifen, trastuzumab, and paclitaxel, can be improved by rosemary extract [194]. These could be promising areas for natural chemical study in the future and a viable breast cancer therapy alternative. 

Although chemotherapy is the most common and effective cancer treatment, chemoresistance, or the inability to respond to chemotherapy, is rising [195]. The activity of ATP-binding cassette (ABC) transporters, which move anticancer medicines out of cells, may influence resistance. As a result, some researchers are attempting to discover natural chemicals that can aid in the reduction of multidrug resistance. Elemene (α-, β-, γ-, and δ-elemene) is a natural chemical that inhibits MDR in MCF-7 and doxorubicin-resistant MCF-7 cells, suggesting that it could have powerful action against multidrug resistance [196]. Another study found that DIM can be used as a radiosensitizer in multidrug-resistant breast cancer cells, allowing it to treat the disease [197]. However, more research on organic chemicals is needed to establish their mechanism of action and possible role in MDR treatment due to the lack of therapeutic targets and the few treatment options. As a result, scientists are working to find novel targets for TNBC and alternative treatments. A wide range of organic complexes was scrutinized, with some generating encouraging outcomes.

Curcumin and resveratrol have been studied [193,194], and both medicines have been shown to effectively cure TNBC with minor adverse effects [195]. The natural component EGCG, which is derived from green tea, has been proven to decrease the migratory capacity of triple-negative breast cancer [196]. A recent study demonstrated that carnosol (phenolic diterpene), a naturally occurring molecule, may arrest the cell cycle in the G2 phase and promote ROS-dependent apoptosis and beclin-1-independent autophagy in triple-negative MDA-MB-231 human breast cancer cells [197]. This shows that carnosol can be utilized to create a TNBC therapy. Natural compounds may therefore be employed to treat TNBC [13]. Some of these compounds may have synergistic impacts or aid in the fight against multidrug resistance. Natural compounds may play an important role in treating and preventing BrCa in the not-too-distant future, given all of these factors. 

## 8. Concluding Remarks

BrCa can be treated with natural chemical compounds generated from living organisms [8]. Raw food ingredients have a long history of use in traditional medicine and can be used in various ways. Research into increasing the action of these chemicals and developing them as a therapy for patients with breast cancer should be prioritized. These natural compounds can be utilized to boost the activity of other common medicines and can also be employed as a therapy system on their own due to their ability to change several pathways. Moreover, they play an essential function in breast cancer prevention. They can function through various methods without unusual negative consequences [13]. Several natural compounds have therapeutic benefits by reversing drug resistance and targeting different targets [10]. A study was conducted to see which parts of BrCa research could have the most impact on BrCa patients if they were focused [198]. Knowledge gaps in BrCa treatment, including natural component absorption, bioavailability, initiation, progression, genetic changes, targets, and diagnostic markers, are currently well recognized [198].

On the other hand, natural substances have been shown through multiple investigations to decrease carcinogenesis and reverse cancer growth by triggering apoptosis and cell cycle arrest. They impact tumor cells by interfering with cell death pathways such as extrinsic and intrinsic apoptosis and autophagy [11]. These compounds inhibit cancer cell proliferation through these processes while causing minimal harm to normal cells [199]. Natural compounds are currently being explored in clinical practice because of their anticancer and apoptotic effects and low toxicity. Many of these substances will likely be used to treat BrCa as they have previously been found to have significant effects against various illnesses [12]. Finally, the natural compounds mentioned are just a fraction of the many chemicals that have been revealed to have anti-BrCa properties. Through the potential of these compounds, researchers are getting closer to finding a cure for BrCa. These compounds have the potential to lower BrCa-related mortality and help people live longer across the world. Therefore, natural substances should continue to be investigated as an option for BrCa therapy.

## Figures and Tables

**Table 2:** Natural chemical combinations used for BrCa therapy and their mode of action.

Combinations and Their Classes	Chemical Formulas	Mode of Action	References
Tetrandrine (alkaloid) and Arsenic (metalloid)	C_38_H_42_N_2_O_6_, and H_3_AsO_4_	Increases FOXO3a, p21, and p27 expression; suppresses cyclin D1 expression; induces G0/G1 phase arrest; promotes autophagy. Survivin expression is also reduced	[183,184]
Curcumin (flavonoid) and Berberine (alkaloid)	C_21_H_20_O_6_, and C_20_H_18_NO_4_^+^	Activates ERK pathways, promoting caspase-dependent apoptosis; induces autophagy; increases JNK and beclin1 phosphorylation; decreases Bcl-2 phosphorylation	[185]
Thymoquinone (1,4-benzoquinone) and Tamoxifen (triphenylethylene)	C_10_H_12_O_2_, and C_26_H_29_NO	Reduces relapse rates, TNF-α, IL-6, and TGF-1β levels; upregulates caspase-3 expression; downregulates Bcl-2 expression; inhibits cell survival via the PI3-K/Akt pathway by suppressing Akt phosphorylation; stimulates XIAP degradation; activates caspase-9, and promotes apoptosis via the PI3-K/Akt pathway; inhibits cell survival by suppressing Akt phosphorylation	[186]
Silibinin (flavonoid)and Chrysin (flavonoid)	C_25_H_22_O_10_, and C_15_H_10_O_4_	Stops proliferation of BrCa cells, and reduces the expression of hTERT and cyclin D1 mRNA	[187]
Resveratrol (phytoalexin)and Salinomycin (polyketide and a spiroketal)	C_14_H_12_O_3_, and C_42_H_70_O_11_	Reduces Wnt signaling protein synthesis, increases E-cadherin and lowers vimentin, slows cell migration and invasion, activates caspase-8 and 9, and downregulates Wnt/EMT signaling	[188]
Garcinol (polyisoprenylated benzophenone) and Paclitaxel (alkaloid)	C_38_H_50_O_6_, and C_47_H_51_NO_14_	Promotes cell cycle arrest, inhibits the (NF-κB)/Twist-related protein 1 (Twist1) signaling system, and suppresses the caspase-3/cytosolic Ca^2+^-independent phospholipase A2 (iPLA2) signaling pathway	[189]
Honokiol (neolignan biphenols) and Lapatinib (4-anilinoquinazoline)	C_18_H_18_O_2_, and C_29_H_26_ClFN_4_O_4_S	Inhibits tumor cell proliferation by suppressing HER-2 expression	[190]

## Data Availability

Available data are presented in the manuscript.
